# Deep learning-based quantification of abdominal fat on magnetic resonance images

**DOI:** 10.1371/journal.pone.0204071

**Published:** 2018-09-20

**Authors:** Andrew T. Grainger, Nicholas J. Tustison, Kun Qing, Rene Roy, Stuart S. Berr, Weibin Shi

**Affiliations:** 1 Departments of Biochemistry & Molecular Genetics, University of Virginia, Charlottesville, Virginia, United States of America; 2 Radiology & Medical Imaging, University of Virginia, Charlottesville, Virginia, United States of America; University of Craiova, ROMANIA

## Abstract

Obesity is increasingly prevalent and associated with increased risk of developing type 2 diabetes, cardiovascular diseases, and cancer. Magnetic resonance imaging (MRI) is an accurate method for determination of body fat volume and distribution. However, quantifying body fat from numerous MRI slices is tedious and time-consuming. Here we developed a deep learning-based method for measuring visceral and subcutaneous fat in the abdominal region of mice. Congenic mice only differ from C57BL/6 (B6) Apoe knockout (Apoe^-/-^) mice in chromosome 9 that is replaced by C3H/HeJ genome. Male congenic mice had lighter body weight than B6-Apoe^-/-^ mice after being fed 14 weeks of Western diet. Axial and coronal T1-weighted sequencing at 1-mm-thickness and 1-mm-gap was acquired with a 7T Bruker ClinScan scanner. A deep learning approach was developed for segmenting visceral and subcutaneous fat based on the U-net architecture made publicly available through the open-source ANTsRNet library—a growing repository of well-known neural networks. The volumes of subcutaneous and visceral fat measured through our approach were highly comparable with those from manual measurements. The Dice score, root-mean-square error (RMSE), and correlation analysis demonstrated the similarity between two methods in quantifying visceral and subcutaneous fat. Analysis with the automated method showed significant reductions in volumes of visceral and subcutaneous fat but not non-fat tissues in congenic mice compared to B6 mice. These results demonstrate the accuracy of deep learning in quantification of abdominal fat and its significance in determining body weight.

## Introduction

Obesity is excessive fat accumulation in the body to the extent that an individual has increased risk for an array of chronic diseases, including type 2 diabetes (T2D), cardiovascular diseases, and cancer [1]. It is a growing epidemic in the US and globally. In 2014, 37.7% of adults and 17% of youth aged 2–19 years were obese in the US [2], and 650 million adults aged 18 years or older were obese worldwide (http://www.who.int/mediacentre/factsheets/fs311/en).

The diagnosis of obesity has primarily relied on a few anthropometric measurements, such as body mass index (BMI), waist circumference, or waist-to-hip ratio. A BMI of ≥ 30 kg/m^2^ is considered obese, and a BMI of 25 to <30 kg/m^2^ is defined as overweight. However, these indirect measurements neither allows for distinguishing fat from skeletal muscle nor distinguishing between visceral and subcutaneous fat. Excessive body fat rather than skeletal muscle is related to both increased plasma levels of free fatty acids and proinflammatory cytokines as well as endoplasmic reticulum stress, all of which contribute to development of insulin resistance, type 2 diabetes, and atherosclerosis [3]. Central or abdominal obesity has been shown to be more closely associated with risk of coronary artery disease and type 2 diabetes [4][5]. Thus, there is a medical demand for accurately measuring the amount and distribution of body fat to better understand its impact on health and disease.

Such imaging modalities as magnetic resonance imaging (MRI) and computed tomography (CT) can clearly distinguish fat from other tissues and thus allow for accurate measurement of fat and non-fat tissue amounts [6]. Compared to CT, MRI involves no ionizing radiation and is more efficient in differentiating fat from non-fat tissues [7][8]. Quantification of body fat volume using MRI or CT involves analysis of many cross-sectional or longitudinal slices across the region of interest. Thus, manual measurement of fat volume with MRI or CT images is a tedious and time-consuming task. To save time and also reduce subjective influences from observers, several semi-automated algorithms have been developed for quantifying body fat [9][10][11][12]. However, nearly all of the algorithms are still dependent on expert knowledge for tuning the features of images and their accuracy and reliability are often low.

Due to recent successes, deep learning with convolutional neural nets has gained popularity in the literature for tackling problems in the areas of image recognition, classification and segmentation [13]. Development of deep learning algorithms relies on neural networks, a computational architecture that can be trained with a large number of annotated images or data, identify features from them, and make predictions of other data. ANTsRNet is a collection of deep learning architecture of neural networks ported to the R language and built on the Keras neural network [14]. Here, we applied these architectures and other open-source software packages developed by the Advanced Normalization Tools team (which includes one of the co-authors) to provide a comprehensive protocol for automatically segmenting abdominal visceral and subcutaneous fat of mice on MR images. The trained networks provided publicly can be directly applied to these MR data for conducting objective and expedited measurements of fat volume in mice.

## Materials and methods

### Ethics statement

All procedures were performed in accordance with the current National Institutes of Health guidelines (https://grants.nih.gov/grants/olaw/Guide-for-the-Care-and-use-of-laboratory-animals.pdf) and approved by the Institutional Animal Care and Use Committee of the University of Virginia (Assurance #A3245-01, Animal Protocol #3109).

### Animals

Apoe knockout (Apoe^-/-^) mice with a C57BL/6 (B6) genetic background were purchased from the Jackson Laboratory. Congenic mice, which only differ from B6-Apoe^-/-^ mice in chromosome 9 region from 15.6 to 115.6 Mb, were constructed using the classical congenic breeding method as previously described [15]. The donor chromosome 9 region was derived from C3H/HeJ (C3H) mice. Mice were fed 14 weeks of Western diet (TD88137, Envigo), stating at 6 weeks of age.

### Magnetic resonance imaging (MRI)

MRI for the abdominal region, which extended from the top of the diaphragm to the bottom of the pelvic cavity, was performed on a 7T Clinscan system (Bruker, Ettlingen, Germany). Mice were anesthetized under isoflurane inhalation during imaging and respiratory motion monitored with an MR-compatible gating system for mice (SA Instruments, Inc., Stony Brook, NY). Axial and coronal T1-weighted sequencing (20 axial and 18 coronal slices; axial voxel size = 0.156×0.156×1.000 mm; coronal voxel size = 0.430×0.430×1.000 mm) at 1-mm-thickness and 1-mm-gap was acquired. Axial 2D images were acquired once with and once without water filtration, and coronal imaging was performed only under the water filtration mode.

### Manual measurement

The volumes of visceral and subcutaneous fat as well as non-fat tissues were quantified on water-filtered axial slices using the auto-thresholding function of the Fiji package for ImageJ [16]. Briefly, MR images (all 35 x 35 mm; Pixel width/height = 0.182291; Voxel depth = 2.0) were converted to binary ones with fat being white and non-fat black, and the “Analyze Particles” function was then used to determine the total white area (mm^2^) on the binary images. The volume of fat (mm^3^) was obtained by multiplying each slice’s total area (mm^2^) by 2 (1-mm slice thickness +1-mm interslice gap), and the total fat volume was the sum of the fat volumes measured from all consecutive slices. The manual measurement results were used as the gold standard for comparisons with the automated measurement results.

### Automatic measurement

**Fig 1:**
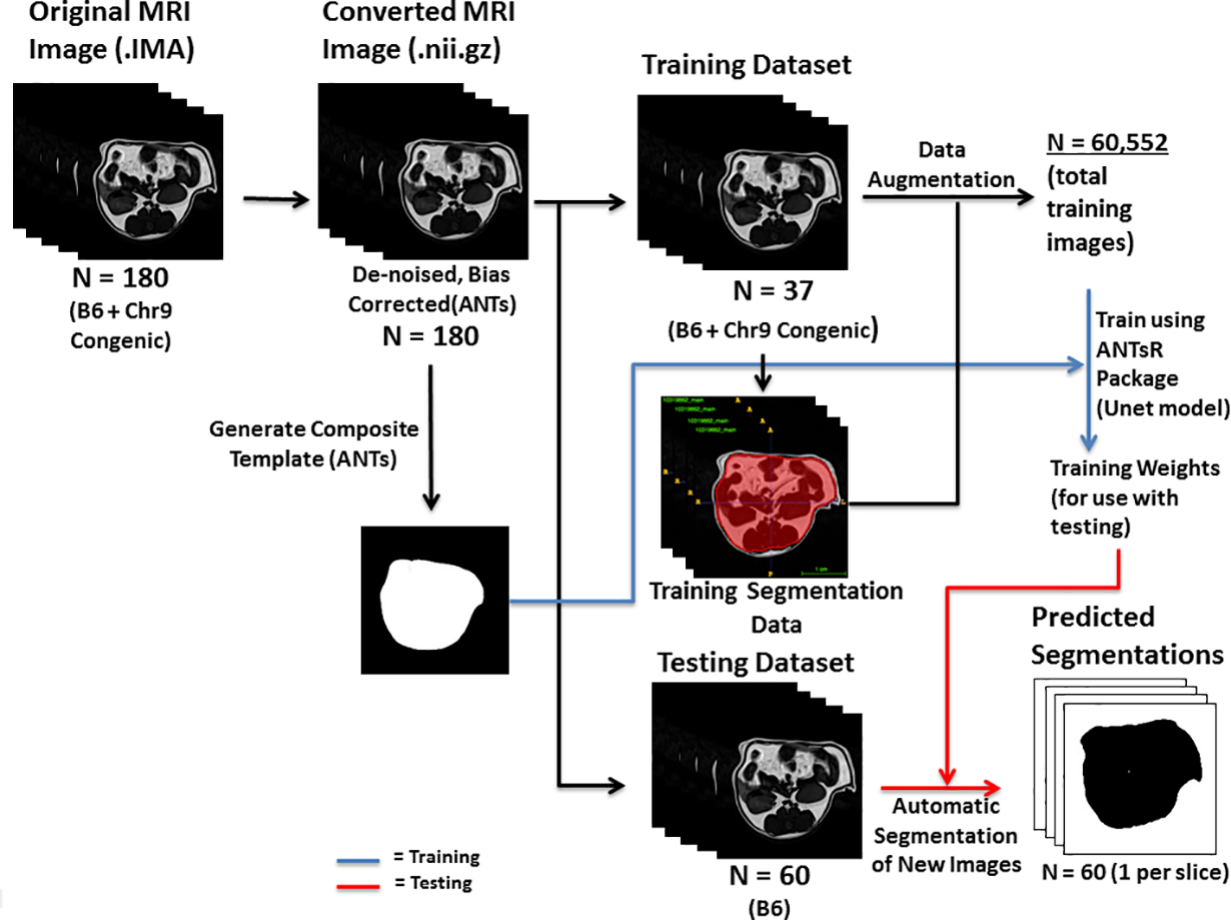
Flowchart illustrating the major steps employed in segmentation and quantification of subcutaneous and visceral fat in MR images. MR images were first converted to a format compatible with the ANTs software packages (NifTI; .nii.gz), and then processed for denoising and bias correction. 37 representative original MR images and their corresponding segmentation images from B6 mice and congenic mice were used for establishing the core training dataset. A novel data augmentation strategy was used to increase the number of training images from 37 to 60,552, which were used to train a U-net-based architecture. A testing dataset consisting of 60 MR images from 3 B6 mice was run through the model to test for accuracy of the method.

The automated method for body fat quantification consists of steps, including training data preparation, template-based data augmentation, and fat quantitation. The complete flowchart of the process is shown in Fig 1.

### Training data preparation

Original MR images were converted to the Nifti format and underwent denoising [17] and bias correction [18] as preprocessing steps to improve image quality. The fat tissue compartment was segmented into subcutaneous and visceral fat based on the peritoneal wall on each image. Visceral fat is the fat within the contour of the abdominal wall, and subcutaneous fat is the fat within the abdominal wall covering the abdominal muscles. Manual labeling was performed using the open source segmentation tool [19]ITK-SNAP [19]. 37 water-filtered 2D MR images from 5 B6 and 3 congenic mice were each manually segmented with fully annotated area for visceral fat and area for subcutaneous fat and these images formed the core training data set. Of them, 26 images were obtained from B6 mice and 11 from congenic mice. These 2D images had a dimension of 35 x 35 x 1 mm with a 1mm gap between consecutive images. They were selected as a diverse and representative set of images spanning the entire abdominal region and varying in intensity and fat amount.

A composite binary mask template was then generated from the segmentation maps of the core training set using the ANTs toolkit (https://github.com/ANTsX/ANTs) [20]. The use of the manually edited binary masks over the original MR images is due to the lack of correspondence of internal structures within the template cohort and our interest in capturing the global shape variation between the visceral and subcutaneous fat regions for the purposes of data augmentation (explained in the next sub-section).

### Template-based data augmentation

The need for large training data sets is a well-known limitation associated with deep learning algorithms [21]. Whereas the architectures developed for such tasks as the ImageNet competition have access to millions of annotated images, such data access is not always available and such is typically the case in medical imaging. In order to achieve data set sizes necessary for learning functional models, various data augmentation strategies have been employed, including application of intensity transformations, such as brightening and enhanced contrast, and simple spatial transformations, such as arbitrary rotations and translations. Regarding the latter, such transformations are not ideal as they might not reflect what is typically seen in medical images and might not sufficiently sample the shape-space of the population currently being studied for generalizability.

**Fig 2:**
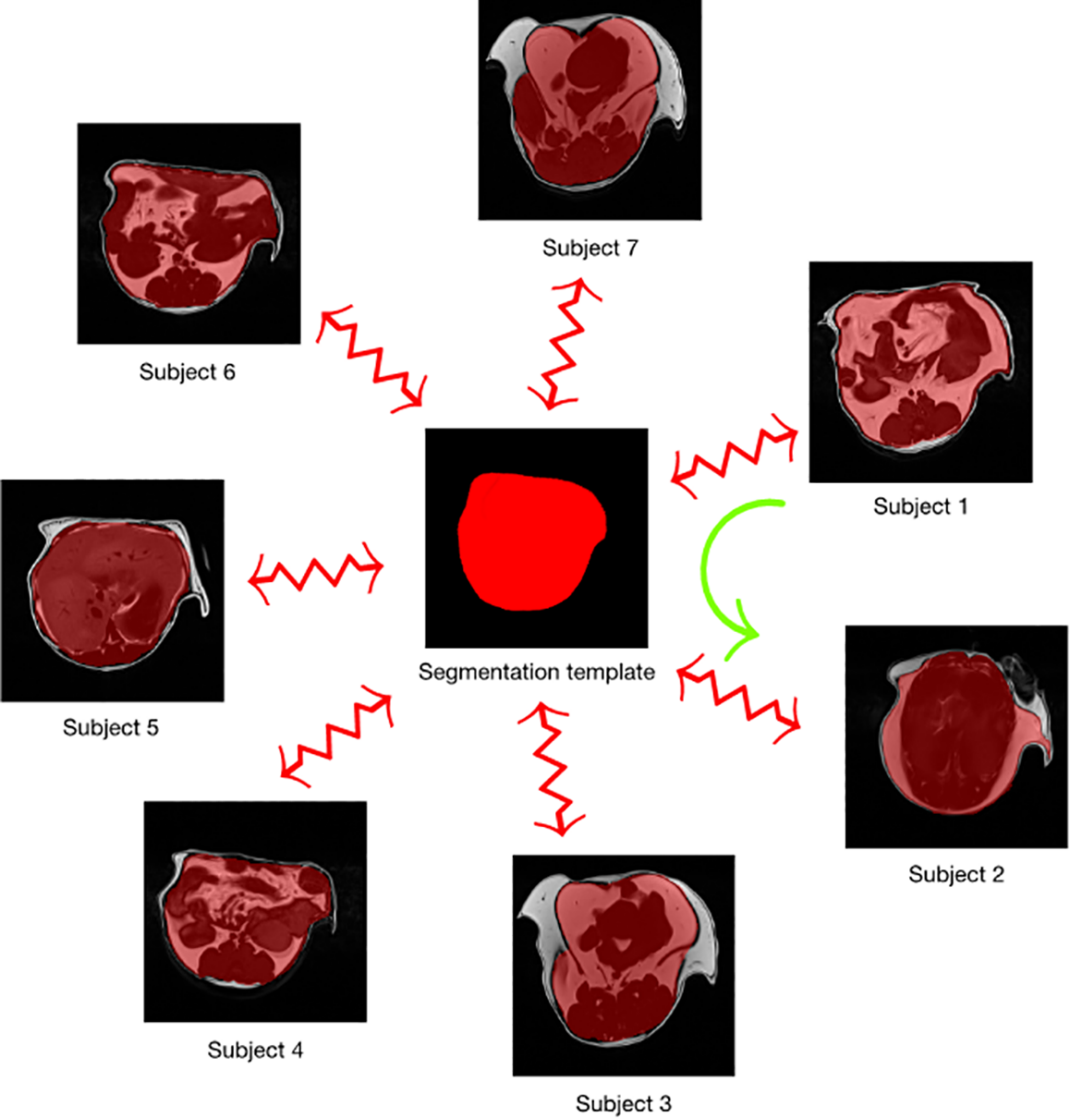
Example MR images showing data augmentation strategy. We introduced a novel data augmentation strategy for training through ANTs-based template construction. A template was created from the training data segmentation images where the red area includes visceral fat and the foreground designates regions of subcutaneous fat. 37 images were used to create such a template that permitting 37^2^ = 1369 possible deformable shapes which are further augmented by random horizontal flipping and randomized rotation.

In this work, we used a template-based approach whereby image data sampled from the population was used to construct a representative template that was optimal in terms of both shape and intensity [20]. In addition to the representative template, this template-building process yields the transformations to/from each individual image to the template space (Fig 2). This permits a propagation of the training data to the space of each individual image. In the simplest case, the training data was used to construct the template and then each individual training data was propagated to the space of every other individual training data. In this way, a training data set of size *N* can be expanded to a data set of size *N*^2^. A more complicated use case could build a template from *M* data sets (where one would expect *M* > *N*). Transformations between the training data and the template could then be used to propagate the training data to the spaces of the individual members of the template-generating data for an augmented data set size of *M x N*.

We used 37 MR images thus permitting 37^2^ = 1,369 possible deformable shapes which comprise the first level of augmented data that can be further augmented by more conventional strategies (e.g., brightness transformations, translations, etc.). The second level of augmented data created two horizontal flip images per level one image as well as 36 rotation images per flip image. In this way we augmented the 1,369 first-level images to 98,568 second-level images (1,369 * 2 * 36).

We implemented tailored batch generators to implement this data augmentation strategy and to generate the data batches per epoch on the fly. Although slower, this avoids the problem of loading all training data in memory during learning. Specific parameters from training can be found in the scripts we wrote for the project available in our bitbucket.org repository [https://bitbucket.org/atg3qz/unet_fat_mri/src].

### U-net for segmentation of abdominal fat in MRI

U-net is a well-known convolutional neural network architecture for voxelwise classification labeling [https://arxiv.org/abs/1505.04597]. It has been employed in various segmentation applications such as MRI of the knee [22], brain tumors in PET imaging [23], and histology images [24]. We combined U-net with our template-based data augmentation scheme described in the previous section for segmenting abdominal fat and subsequent quantification.

### ANTsRNet, an open-source repository for deep learning architectures

We also introduced ANTsRNet [https://github.com/ntustison/ANTsRNet], a collection of well-known deep learning architectures ported to the R language. ANTsRNet is built using the Keras neural network library [14] (available through R) and is highly integrated with the ANTsR package, the R interface of the ANTs toolkit. Consistent with other ANTs-based software offerings, ongoing development is currently carried out on GitHub using a well-commented coding style, thorough documentation, and self-contained working examples. One such architecture is the well-known U-net architecture [https://arxiv.org/abs/1505.04597] where we have replaced the cross-entropy loss function with a multi-label Dice function based on previous work [http://hdl.handle.net/10380/3141].

Input and testing nifti images are all of size 192 × 192, as is the template, such that no resampling is required. I/O from the disk to the ANTsRNet software is handled by antsReadImage/antsWriteImage functions available as part of ANTsR. During data augmentation, the antsApplyTransforms program was used to transform a randomly chosen image/segmentation mask pair to a randomly chosen target in the training cohort. During data augmentation, a digital “coin toss” was used to randomly flip the image/segmentation pair in a left-right direction followed by a randomly chosen rotation angle between 0 and 359 degrees. These latter operations were handled by the magick package in R. Specific parameters for the 2-D U-net architecture for both models are as follows:

- Adam optimization:
  ○ learning rate = 0.0001.
  ○ optimization function: Dice coefficient.
- Number of epochs: 40.
- Convolution layers:
  ○ -kernel size: 3×3.
  ○ -activation: rectified linear units (ReLU).
  ○ -number of filters: doubled at every layer starting with N = 32.
- Max pooling layers:
  ○ size: 2×2.
  ○ stride length: 2×2.
- Upsampling/transposed convolution (i.e., deconvolution) layers:
  ○ kernel size: 2×2.
  ○ stride length: 2×2.
  ○ activation: rectified linear units (ReLU).

Training took approximately 1.5 hours. After model construction, prediction per image (after preprocessing) takes < 1 second per image. Both model construction and prediction utilized a NVIDIA Titan Xp GPU. Both training and prediction are handled by custom-built R scripts available as part of the Bitbucket repository associated with this work.

### Fat quantification with segmented images

**Fig 3:**
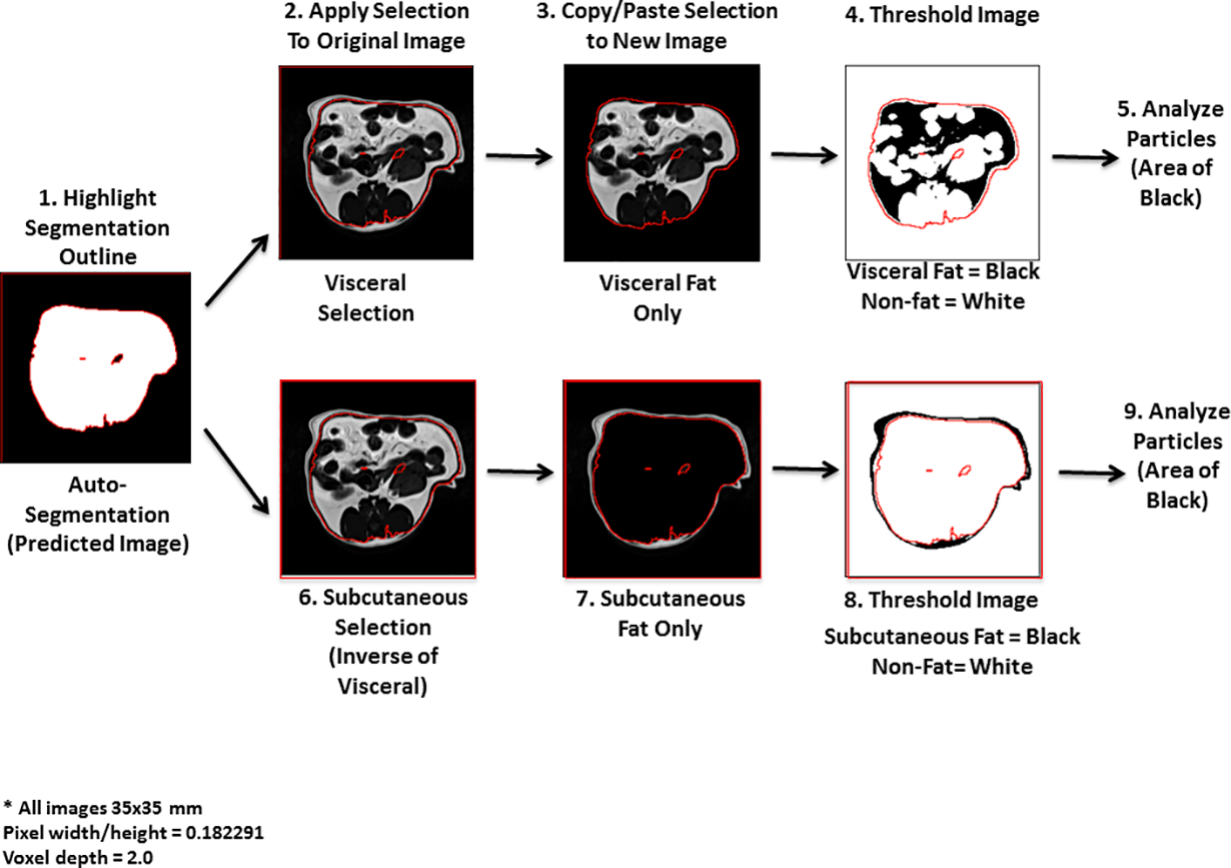
Flowchart showing major steps in automated quantification of visceral and subcutaneous fat. Images were created using the Fiji package of ImageJ. All MR images were set to the dimensions of 35x35 mm, a pixel width and height of 0.182291, and a voxel depth of 2. 1) Make the visceral segmentation image binary (outside = black, visceral component = white), and use the “Create Selection” tool to generate a selection perfectly outlining the visceral component. 2) Use the ‘Restore Selection” tool to place this selection on the original MR image, giving an exact outline of the visceral component. 3) Copy and paste this selected area to a new image, making sure it is the same dimensions as the original MR image to ensure accurate quantification (all images 35 x 35 mm; Pixel width/height = 0.182291; Voxel depth = 2.0). 4) Threshold the image to make a binary image in which the fat is one color and everything else is another. 5) Quantify this new image by using the “Analyze Particles” tool. 6) On the original MR image with the visceral fat component selected (from 2), use the “Make Inverse” tool to obtain the subcutaneous fat component. 7–9) Repeat the same quantification process as seen in 3–5.

The novel segmentation images were evaluated for accuracy for quantitation of visceral and subcutaneous fat using the Fiji package. To measure visceral fat, the segmentation image was used as a template to generate a contour, which was then overlapped onto the original MR image to outline the region containing visceral fat (Fig 3). The outlined area was made binary with visceral fat to the black color and other components to white. The “Create Selection” tool was then used to generate a contour perfectly outlining the visceral fat component. The ‘Restore Selection” tool applied to place this selection onto the original MR image, giving an exact outline of the visceral component in the image. This selected area was copied and pasted to a new image (all images 35 x 35 mm; Pixel width/height = 0.182291; Voxel depth = 2.0), and the area of the image was measured in the same manner as described above under “Manual measurement”. To quantify subcutaneous fat, the “Make Inverse” tool was used to select the inverse of the visceral component and the selected area was again pasted to a new image.

### Statistical analysis

Comparisons were made between the automated and manual methods in quantification of visceral and subcutaneous fat as well as between B6 and congenic mice for differences in body weight and fat volume. The Dice score, generated from the ANTs Utilizes Software package, and the root-mean-square error (RMSE) were used for comparing the similarity of the values of fat volumes measured by the manual and automatic methods in the same set of MR images. Correlation analysis was performed to determine the correlation of visceral or subcutaneous fat volumes measured by the two methods. For comparisons of fat volumes between congenic and control mice at multiple slices, two-way ANOVA was conducted. When the F value was significant (*P* < 0.05), Student's t-test was performed to determine differences between the groups. T-test was also used to determine the difference in body weight between the 2 groups of mice. Differences were considered statistically significant at *P* < 0.05.

## Results

### Phenotypic difference in body weight

**Fig 4:**
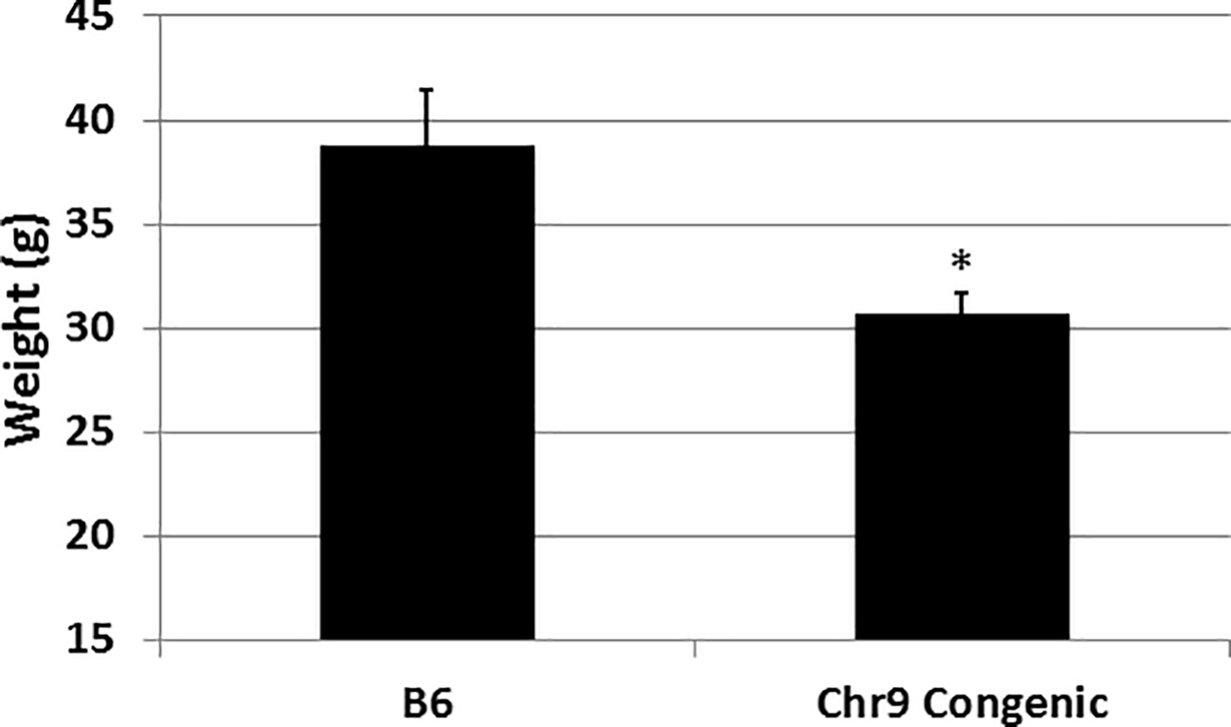
Body weight (g) of male congenic and B6-Apoe^-/-^ mice fed a Western diet. Results are means ± SE of 7 B6 and 17 congenic mice after 14 weeks of Western diet. * *P* < 0.05 vs. B6 mice.

Chromosome 9 congenic mice were genetically identical to B6-Apoe^-/-^ mice except for the chromosome 9 region (15.6–115.6 Mb), which was replaced with the C3H genome [15]. After being fed 14 weeks of Western diet, male congenic mice displayed significantly lighter body weight than B6-Apoe^-/-^ mice (30.67 ± 1.05 vs. 38.81 ± 2.66 g; *P* = 0.0021; n = 7 and 17, respectively) (Fig 4). This result confirmed the existence of *W10q13*, a locus for body weight on mouse chromosome 9 we previously mapped in an intercross derived from B6-Apoe^-/-^ and BALB/cJ-Apoe^-/-^ mice [25].

### Manual Measurement of body fat

**Fig 5:**
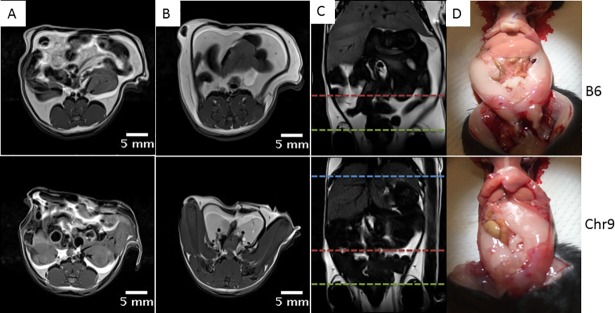
Representative MR images of congenic and B6-Apoe^-/-^ mice fed a Western diet. Axial MR slices at the levels denoted by the red (A) and green lines (B) across the coronal slice (C). D, gross examination of abdominal fat. Tope roll: B6-Apoe^-/-^ mice; bottom: congenic mice.

**Fig 6:**
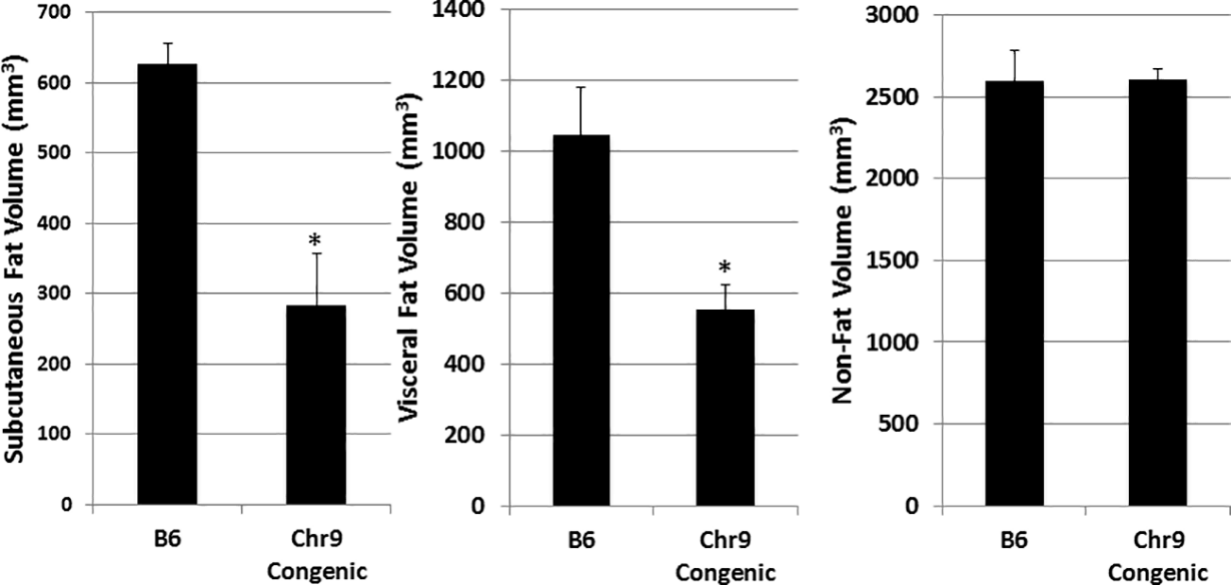
Fat and non-fat volumes of congenic and B6-Apoe^-/-^ mice measured manually using axial MR slices. Results are means ± SE of 4 mice per group. * *P* = 0.012.

MRI scans were performed within one week before mice were euthanized. Noticeable differences in subcutaneous and visceral fat amounts between congenic and B6-Apoe^-/-^ mice could be seen on MR images and gross anatomic examination (Fig 5). To quantify the differences, the volumes of subcutaneous and visceral fat were measured on 4 representative slices (1 slice at the level of liver, 2 at kidney, 1 at pelvic) spanning the abdominal region for congenic and B6-Apoe^-/-^ mice (4 mice per group). Congenic mice had a subcutaneous fat volume of 284.0 ± 72.4 mm^3^/slice, significantly smaller than the volume of 627.5 ± 29.9 mm^3^/slice in B6-Apoe^-/-^ mice (p = 0.0046) (Fig 6). The visceral fat volume was also significantly smaller in congenic mice (554.6 ± 70.3 mm^3^/slice) than in B6 mice (1045.6 ± 136.4 mm^3^/slice) (p = 0.019). In contrast, non-fat tissue volume was comparable between congenic and B6-Apoe-/- mice (2603.8 ± 66.6 vs. 2597.7 ± 189.5 mm^3^/slice).

### Automatic measurement of visceral and subcutaneous fat

**Fig 7:**
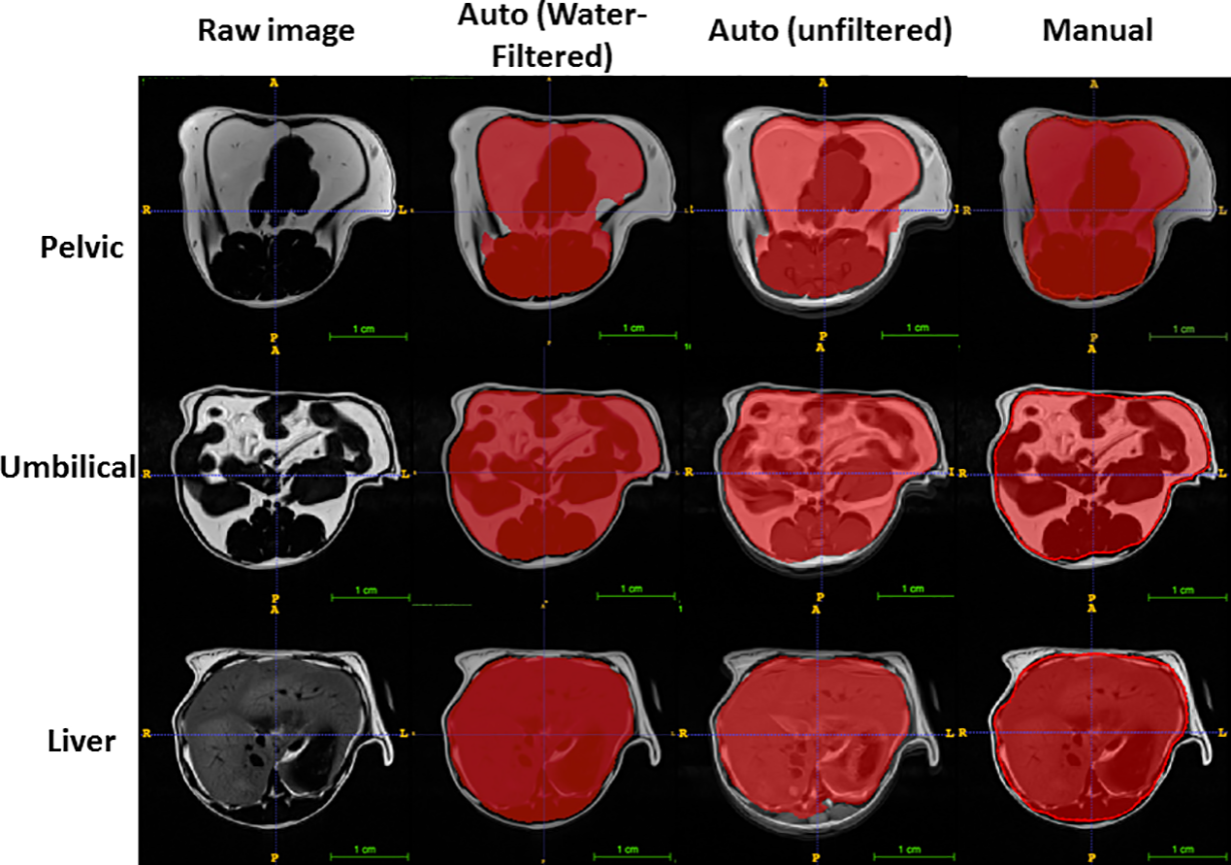
The accuracy of deep learning in deriving the area containing visceral fat at three different levels: pelvic, kidney and liver on MR images. Prediction of the segmentation in the red area by deep learning is highly consistent with the input data. The red area is where visceral fat is included. Auto: prediction made by deep learning; manual: the red line is drawn with ImageJ and the area within the red line is used as input for visceral fat.

**Fig 8:**
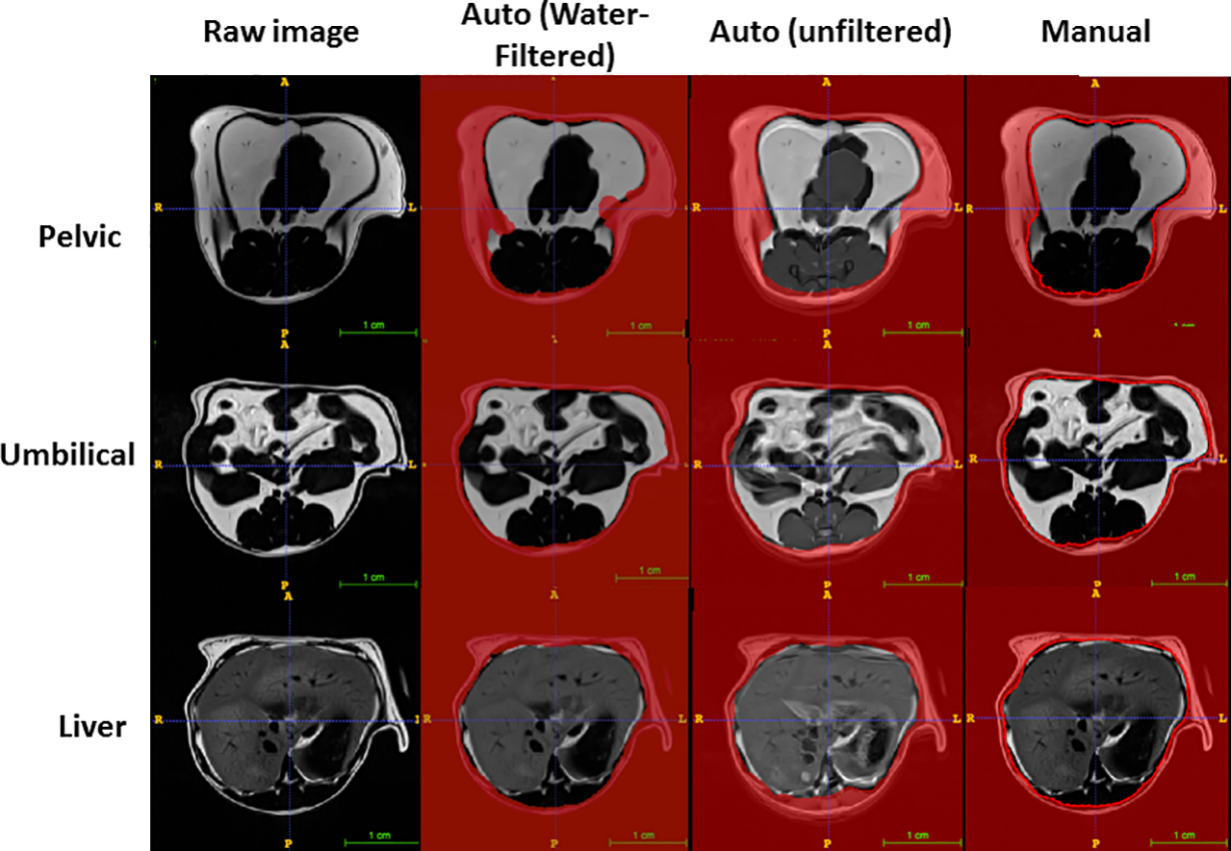
The accuracy of deep learning in deriving the area containing subcutaneous fat at three different levels: Kidney and liver on MR images. The red area is where subcutaneous fat is located. Auto: prediction made by deep learning; manual: the red line is drawn with ImageJ and the area outside the red line is used as input for subcutaneous fat.

A modified deep learning approach with a novel template-based data augmentation strategy was employed. The U-net model was trained with axial MR images spanning the entire abdominal region of mice. We then applied the learned U-net model to analysis of 60 water-filtered evaluation MR images from three B6 mice that were previously quantified manually to generate corresponding probability image sets designating subcutaneous and visceral regions. Automatic segmentations of visceral and subcutaneous fat for either water-filtered or unfiltered slices generated from corresponding probability images were highly consistent with the manually generated segmentations of the same input images (Figs 7 and 8). These segmentation images were then used to generate the regions of interest on the original MR slices for quantifying both visceral and subcutaneous fat volumes.

**Fig 9:**
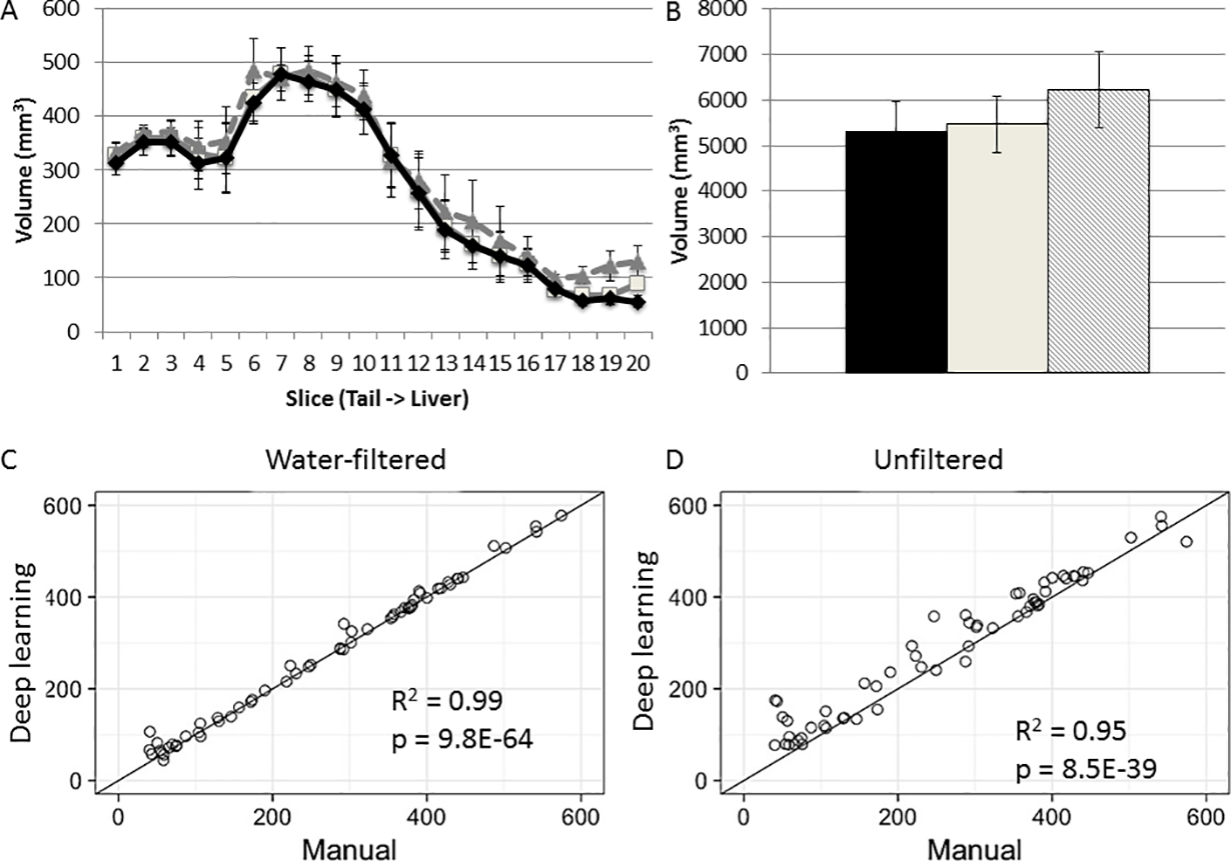
Comparison between the automated and manual measurements in quantification of visceral fat. **A,** The volumes of visceral fat on 20 sequential axial slices from the tail root (slice 1) to the diaphragm (slice 20) of B6-Apoe^-/-^ mice measured by manual (black) and deep learning (water filtered = solid; unfiltered = dashed). **B,** the total fat volume of B6-Apoe^-/-^ mice measured by manual (black) and deep learning (grey; water filtered = solid, unfiltered = dashed). **C**, Correlation analysis of visceral fat volumes on water-filtered MR slices measured with the two methods. Each dot represents on axial MR slice. R^2^ and P values are shown in the figure. **D,** Correlation analysis of visceral fat volumes on unfiltered MR slices measured with the two methods.

Comparative analyses were performed to verify the accuracy of the automated method through multiple metrics, including Dice’s similarity coefficient, RMSE, correlation coefficient, and real measurement results. We analyzed 43 slices that had not been included in the original training set and calculated Dice coefficient values measuring the level of overlap between the manual and automatic segmentation results. The average Dice coefficient value was 0.968 ± 0.00267 (Min = 0.919, Max = 0.987) (S1 Data). The volumes of visceral fat measured from sequential water-filtered slices were comparable between the two measurements (Fig 9A). The total visceral fat volume measured by the automated method was also comparable to the volume achieved by the manual method (5463 ± 625 vs. 5331 ± 642 mm^3^; p = 0.890) (Fig 9B). Correlation analysis showed an extremely high agreement in measurements made by the two methods (R^2^ = 0.99; p = 9.8E-64) (Fig 9C). The RMSE values supported this close correlation (S1 Data).

**Fig 10:**
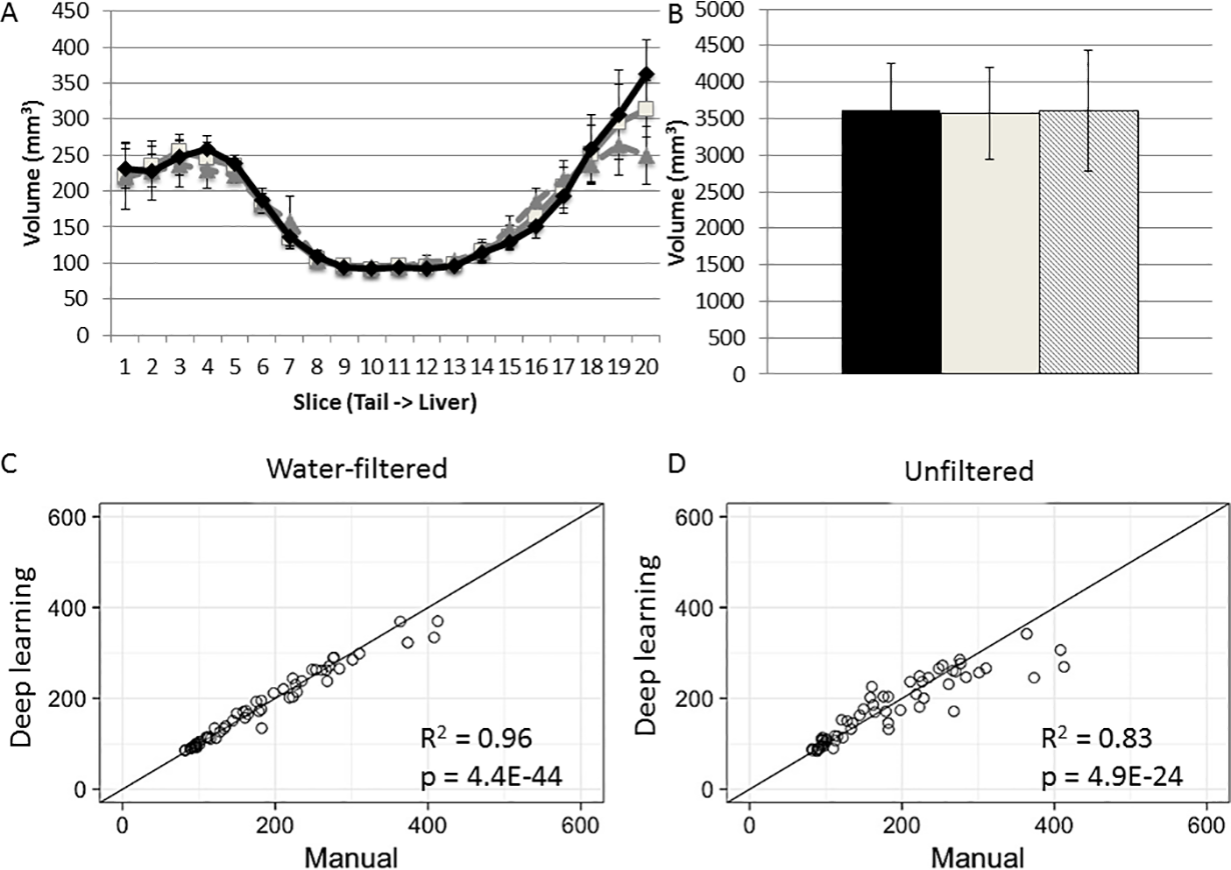
Comparison between the automated and manual measurements in quantification of subcutaneous fat. **A,** The volumes of subcutaneous fat on 20 sequential axial slices from the tail root (slice 1) to the diaphragm (slice 20) of B6-Apoe^-/-^ mice measured by a manual (black) or automated method (water filtered = solid; unfiltered = dashed). **B,** the total subcutaneous fat volume of B6-Apoe^-/-^ mice measured by a manual (black) or automatic method (water filtered = solid; unfiltered = dashed). **C**, Correlation analysis of subcutaneous fat volumes on water-filtered MR slices measured with the two methods. Each dot represents a slice. R^2^ and P values are shown in the figure. **D,** Correlation analysis of subcutaneous fat volumes on unfiltered MR slices measured with the two methods.

The volumes of subcutaneous fat measured on sequential water-filtered slices were also comparable between the two measurements (Fig 10A). The total subcutaneous fat volume from auto-segmentation was also similar to the volume obtained from the manual method (3571 ± 141 vs. 3617 ± 122 mm^3^; *P* = 0.533) (Fig 10B). Correlation analysis shows a high agreement in measurement results achieved from the two methods (R^2^ = 0.96) (Fig 10C).

The automated method also applied to measurement of fat volumes on unfiltered MR slices, though they had not been used to train the algorism. The Dice coefficient for the corresponding unfiltered images still shows a high degree of overlap with manual segmentations with a value of 0.962 ± .00467 (Min = 0.772, Max = 0.990), however the overlap is slightly worse than with water-filtered images. The total visceral fat volume was slightly higher than the volume measured manually on water-filtered slices (6232 ± 834 vs. 5331 ± 642 mm^3^; p = 0.565) (Fig 9). Correlation analysis showed a high degree of correlation between the results achieved from the two measurements (R^2^ = 0.95; p = 8.5E-3(. The total subcutaneous fat volume was 3606 ± 117 mm^3^, comparable to the volume of 3571 ± 141 mm^3^ measured manually on water filtered slices (*P* = 0.816). A significant correlation between the manual measurement made on water-filtered images and the automated measurement made on unfiltered images was observed (R^2^ = 0.8278; p = 4.9E-24). A small mismatch in the measurement results was observed in slices acquired at a higher location where the liver was located, though the difference was not significant.

### Differences between congenic and B6-Apoe^-/-^ mice in subcutaneous and visceral fat

**Fig 11:**
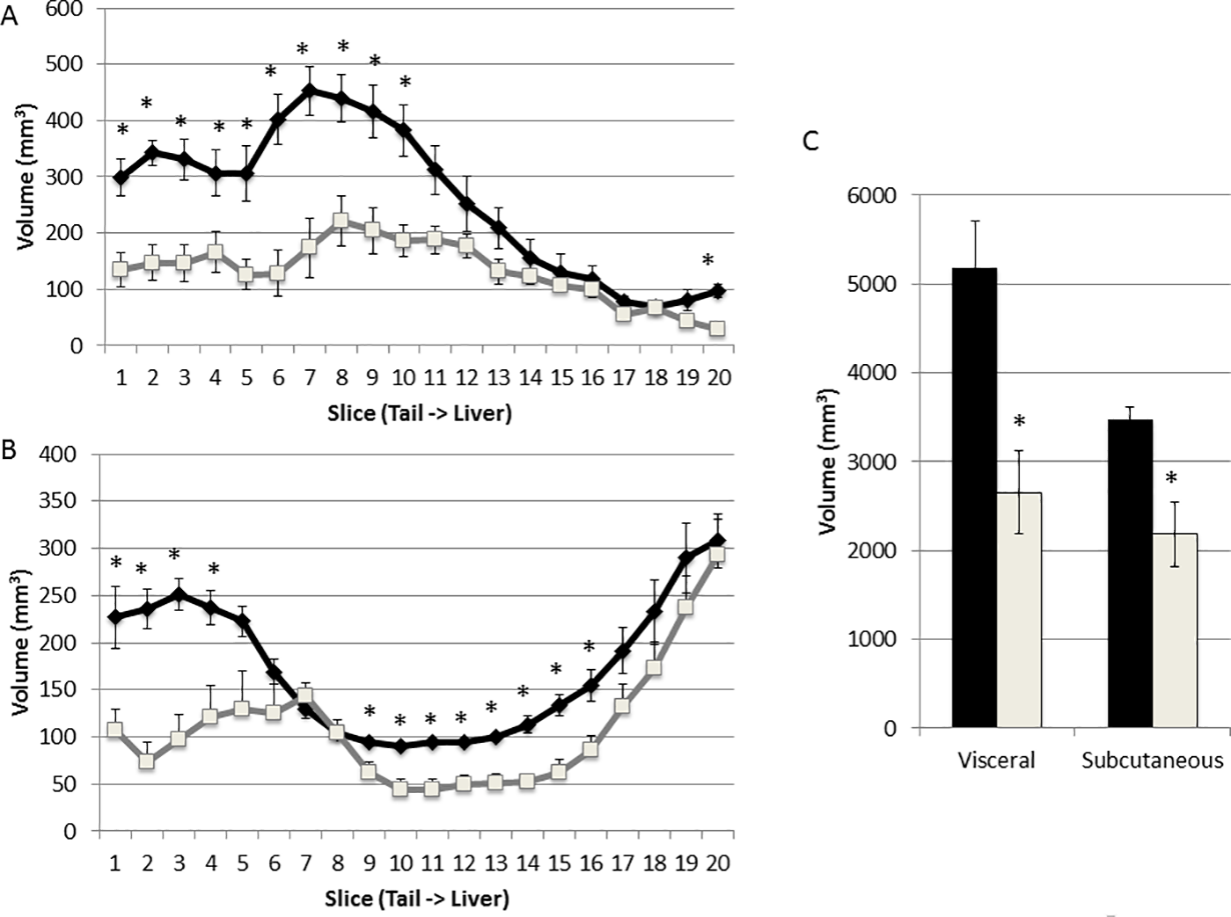
**Comparison between congenic and B6-Apoe**^**-/-**^ **mice in visceral (A) and subcutaneous fat volumes (B) measured by the automated method on water filtered MR slices.** MR slices span from the pelvic cavity (slice 1) to the top of the liver (slice 20). Results are means ± SE of 4 mice per group on each slice. **C,** The total volume of visceral and subcutaneous fat in the abdominal region of congenic and B6-Apoe^-/-^ mice. * P < 0.05.

The automated method was used to measure fat volumes on axial water-filtered slices acquired from congenic and B6-Apoe^-/-^ mice (n = 4 per group). Congenic mice had less subcutaneous and visceral fat than B6-Apoe^-/-^ mice in most slices scanned (Fig 11). The differences were more significant in the lower half of the abdominal region for visceral fat. The total visceral fat volume of congenic mice was reduced by 48.8% relative to the volume of B6 mice (2652 ± 465 vs. 5183 ± 523 mm^3^; p = 0.011). The subcutaneous fat volume of congenic mice was reduced by 37.1% (2184 ± 363 vs. 3471 ± 141 mm^3^; p = 0.016).

## Discussion

An increasing prevalence of central obesity and its close association with cardiovascular disease and type 2 diabetes demands a reliable technique for abdominal fat quantification. MRI is the most effective imaging modality currently used for detecting the fat tissue in vivo, but quantifying fat volume from numerous slices remains a challenge. In this study, we developed a deep learning-based method for measurement of abdominal fat using MR images obtained from two mouse strains that differed markedly in body weight on a high fat diet. Volumes of visceral and subcutaneous fat and non-fat tissues measured by the algorithm have shown a high degree of consistency with those achieved by a manual method. Moreover, we have found that body fat rather than non-fat tissues accounts for the difference between the two strains in body weight.

In this study, we trained the modified U-net network with 37 original MR images spanning the abdominal region of mice. Despite the limited number, the algorithm has shown a better performance than the prior best automatic method for quantification of visceral and subcutaneous fat using MR images [12]. We calculated the Dice coefficient value to determine the overlap between the new algorithm and the manual method in the measurement of visceral fat with the same set of MRI slices. Our results have shown a high similarity between the two methods in quantifying visceral fat, by achieving an average Dice value of 0.968. Pearson's correlation analysis was performed to evaluate the consistency of subcutaneous and visceral fat volumes measured by the two methods. The correlation coefficient of the values obtained from the two methods was 0.99 for visceral fat quantification and 0.96 for subcutaneous fat quantification made on water-filtered MR images. Correlation may not be a perfect measure of similarity because a predictor that consistently predicts half of the actual weight, would yield a correlation coefficient similar to the one when the predictor exactly predicts the actual weight. In contrast, the root mean square error (RMSE) does not suffer from this caveat. The small differences between values from the two methods demonstrate the effectiveness of deep learning in quantifying abdominal fat (S1 Data). To evaluate performances of the algorithm at different segmentation locations, we compared the volume values of visceral and subcutaneous fat on each of the sequential MRI slices as well as the total fat volumes obtained from the two methods. The automatically obtained fat volumes have shown a high consistency with those obtained manually.

We also applied the algorithm to the quantification of visceral and subcutaneous fat volumes on unfiltered MR images acquired from mice. Though the network had not been trained with unfiltered MR images, the results achieved still showed a high level of consistency with those achieved manually for both visceral and subcutaneous fat. Automated segmented images had an average Dice coefficient of 0.962 compared to manually-generated counterparts. The correlation coefficient of the fat volume results on individual slices was 0.95 for visceral fat and 0.83 for subcutaneous fat. However, we did find that the total visceral fat volume had a slightly larger value than the manual measurement and the correlation coefficients of the fat volume results with respect to the comparison between the two measurement methods were smaller relative to those achieved from water filtered MR images. A possible explanation for the gap in fat volume quantification is that the algorithm had not been trained with unfiltered MR images, which have a less clear contrast between the fat and non-fat tissue compared to water filtered MR images. It’s also noteworthy that the automated and manual measurement results were not made from the same MR images but the images from two separate scans at comparable locations.

We observed that adipose tissue rather than non-fat tissues underlies the difference between congenic and B6-Apoe^-/-^ mice in body weight on the Western diet. Indeed, congenic mice had a ~50% reduction in visceral fat volume and ~40% reduction in subcutaneous fat compared to the B6 mice but there was no reduction in non-fat tissue volume. The congenic mice were constructed to verify a locus initially mapped in an intercross between B6 and C3H Apoe^-/-^ mice and then replicated in at least four independent crosses [26][27][28][29]. The congenic strain was highly resistant to atherosclerosis [15]. As obesity is a major risk for atherosclerosis, the reduction in abdominal fat could contribute to the resistance of congenic mice to atherosclerosis. Because congenic mice had reductions in both visceral and subcutaneous fat, this result does not allow for judging which type of fat might contribute to their resistance to atherosclerosis.

In addition, we have found that the pattern of fat distribution differed in the two strains: visceral fat mostly accumulated in the lower half of the abdomen in B6-Apoe^-/-^ mice whereas the visceral fat of congenic mice more evenly distributed in longitudinal direction. B6-Apoe^-/-^ mice showed much more subcutaneous fat accumulation in the distal end of the abdomen compared to congenic mice.

In summary, the use of deep learning can accurately quantify visceral and subcutaneous fat of mice on MR images. The next logical step is to determine whether this new approach can be applied to humans. Nevertheless, because of the anatomical and biochemical similarities between the mouse and the human, we are confident that this algorithm should be applicable to quantification of abdominal fat in humans.

## Supporting information

S1 Data All data in this article. (XLSX)
